# Fetal growth and the risk of childhood non-CNS solid tumours in Western Australia

**DOI:** 10.1038/sj.bjc.6604424

**Published:** 2008-06-17

**Authors:** C L Laurvick, E Milne, E Blair, N de Klerk, A K Charles, C Bower

**Affiliations:** 1 Telethon Institute for Child Health Research, Centre for Child Health Research, The University of Western Australia, PO Box 855, West Perth, Western Australia; 2 Paediatric and Perinatal Pathologist, PathWest, PMH and KEMH Hospitals, Subiaco, Perth WA Clinical Senior Lecturer, Schools of Paediatrics and Child Health, and Women and Infants Health, University of Western Australia, Roberts Road, Subiaco, Western Australia

**Keywords:** childhood cancer, intra-uterine growth, risk factor, Cox regression

## Abstract

Using population-based linked health data, we investigated whether the risk of certain childhood non-CNS solid tumours (*n*=186) was associated with intra-uterine growth. The risk of retinoblastoma and rhabdomyosarcoma, but not other tumour types, was positively associated with increased growth, suggesting a possible role of fetal growth factors. Larger studies are needed.

Some childhood cancers occur more commonly in children below the age of 5 years, suggesting that *in utero* factors may be important in their aetiology. Birth weight has been frequently studied in relation to risk, and is one of the few factors for which positive associations have been reported. However, birth weight studies of childhood non-central nervous system (non-CNS) solid tumors have produced inconsistent results and few have taken account of gestational age to differentiate between an association with birth weight *per se* from one with accelerated intrauterine growth. The mechanism(s) underlying the observed associations has not been well elucidated, but insulin-like growth factors (IGFs) have been implicated (LeRoith *et al*, 1991).

We previously reported an increased risk of ALL (acute lymphocytic leukaemia) (Milne *et al*, 2007), of Hodgkin lymphoma and Burkitt lymphoma among boys, and of non-Hodgkin lymphoma among girls (Milne *et al*, 2008) in association with increasing proportion of optimal birth weight, a measure of the appropriateness of fetal growth (Blair *et al*, 2005). These findings are consistent with a biologically plausible mechanism of accelerated growth associated with growth factors in normal as well as cancer cells. The aim of the present study was to investigate whether the risk of non-CNS solid tumours was also associated with fetal growth.

## Methods

We analysed anonymised, population-based data from the Western Australian Data Linkage System (Holman *et al*, 1999). Among births between 1980 and 2004, eligible subjects comprised 281 cases of non-CNS solid tumours below the age of 15 years and 576 352 non-cases. We used cancer categories based on the International Classification of Childhood Cancer, 3rd Edition (ICCC3); only categories with more than 20 cases were included in the analysis: neuroblastoma/ganglioneuroblastoma (hereafter referred to as ‘neuroblastoma’) (*n*=69), retinoblastoma (*n*=38), Wilms' tumour (*n*=52) and rhabdomyosarcoma (*n*=27).

Explanatory variables included z-scores of ‘proportion of optimal birth weight’ (POBW), ‘proportion of optimal birth length’ (POBL) and ‘proportion of optimal weight for length’ (POWFL) (Blair *et al*, 2005); three measures of the appropriateness of intra-uterine growth (IUG) described previously (Milne *et al*, 2007). Briefly, POBW is the ratio of the observed to the ‘optimal birth weight’; the latter estimated from a regression equation including terms for gestational duration, maternal height, parity and infant sex, derived from a total population of singleton births that excluded those exposed to risk factors for IUG restriction, including maternal smoking. POBL is the comparable measure of the appropriateness of longitudinal growth, and primarily reflects skeletal growth. POWFL is similarly derived and is a measure of the appropriateness of total weight for length and predominately reflects soft tissue growth. POBW, POBL and POWFL were expressed as z-scores, so the risk of a specific tumour was estimated per s.d., allowing direct comparison of regression coefficients. The population means and s.d. used in the calculation of the z-scores for each variable were those shown for the non-case group in Table 1. Data were analysed using Cox proportional hazards regression in STATA version 9.0 (StataCorp, 2005).

## Results

The sex distribution varied by type of tumour, with a higher proportion of males than females with neuroblastoma and retinoblastoma, more than half of which with were diagnosed before 2 years of age. The proportion of first-born children was higher in each tumour group than in the non-case group (Table 1).

The risk of retinoblastoma (HR: 0.54) was lower among girls than boys (Table 2). Overall, there was little evidence of an association between the three IUG measures and risk of neuroblastoma or Wilms' tumour. There appeared to be weak positive associations between POBW and retinoblastoma (HR: 1.22) and rhabdomyosarcoma (HR: 1.33) (Table 2). Similarly, POBL appeared to be positively associated with retinoblastoma (HR: 1.26) and POWFL with rhabdomyosarcoma (HR: 1.41) (Table 2), though few associations were statistically significant.

As in our approach with childhood ALL (Milne *et al*, 2007), CNS tumors and lymphomas (Milne *et al*, 2008), we aimed to distinguish an effect of high birth weight *per se* and one with accelerated growth. We restricted the univariate regression analysis of POBW to, in turn, children with birth weights below two commonly used definitions of high birth weight: >3500 and >4000 g. The positive associations observed between POBW and retinoblastoma and rhabdomyosarcoma were also observed among children with birth weights <3500 and <4000 g: retinoblastoma (HR: 1.39, 95% CI 0.82–2.36 and HR: 1.32, 95% CI 0.89–1.95, respectively); and rhabdomyosarcoma (HR: 1.53, 95% CI 0.82–2.86 and HR: 1.55, 95% CI 1.00–2.40, respectively).

## Discussion

Our measures of the appropriateness of fetal growth – POBL, POBW and POWFL – are independent of gestational age and take account of the major non-pathological determinants of IUG. As with ALL (Milne *et al*, 2007) and lymphomas (Milne *et al*, 2008), there was a positive association between at least one measure of IUG and risk of retinoblastoma and rhabdomyosarcoma. We found no evidence of an association between IUG and risk of neuroblastoma and, unlike previous studies (Leisenring *et al*, 1994; Yeazel *et al*, 1997; Schuz *et al*, 2001), we found no association between IUG and the risk of Wilm's tumour.

Growth is a mixture of skeletal growth – tending to be expressed as increased height; and somatic growth, which may be proportionate (ie, a large child, but with a normal body mass index), or disproportionate (increased soft tissue with a raised body mass index/POWFL). Insulin-like growth factors play a major role in regulating the normal growth and differentiation of cells and tissues during fetal development (LeRoith *et al*, 1991). Some tumours produce IGF-I, IGF-II or their binding proteins, or possess IGF receptors (Campbell and Novak, 1991; Antoniades *et al*, 1992; Hirschfeld and Helman, 1994; Boulle *et al*, 1998). IGF-I, in particular, inhibits the process of programmed cell death in both normal and DNA-damaged cells (Barres *et al*, 1992; Baserga *et al*, 1997a). The mitotic properties of IGFs, coupled with their ability to inhibit cell death, are thought to enhance tumour growth (Baserga *et al*, 1997b; Werner and Le Roith, 2000; Bentov and Werner, 2004; Pollak *et al*, 2004). Different tumours are likely to have different underlying genetic predispositions, which in turn are likely to be reflected in different patterns of growth, which may partly explain the associations we observed.

This study has some important strengths. Examining risk associated with the appropriateness of IUG allows a more detailed exploration of this relationship than using birth weight alone or birth weight with adjustment for gestational age. We were able to explore associations between some specific solid cancers of childhood and three distinct aspects of IUG: POBW, POBL and POWFL. The z-scores for each of these were modeled appropriately as continuous variables and this method obviated the need to assign an arbitrary cutoff for ‘high birth weight’. Being a population-based, record-linkage study, neither selection bias nor recall bias would have affected our results.

There were small numbers of cases in this study and many results were only suggestive of an association; however, our findings are consistent with literature describing biologically plausible mechanisms for associations between increased fetal growth and risk of some non-CNS solid tumours. The persistence of our results when the analysis was restricted to children without high birth weight further supports an association with accelerated growth, rather than high birth weight *per se*.

We recommend for future studies, the use of measures of the appropriateness of IUG rather than birth weight alone, particularly in large collaborative studies that can examine these relationships with greater power.

## Figures and Tables

**Table 1:** Descriptive characteristics of non-CNS solid tumours in children aged 0–14 years in Western Australia

		**Sex**		**Age at diagnosis**		**Birth order**		**POBW**	**POBL**	**POWFL**
	***N***	**Males *N* (%)**	**Females *N* (%)**	**<2 years *N* (%)**	**2+ years *N* (%)**	**First born *N* (%)**	**Subsequent born N (%)**	**Mean (s.d.)**	**Mean (s.d.)**	**Mean (s.d.)**
Non-cases	576352	293766	282586	—	—	295903	280449			
		(51.0)	(49.0)			(51.3)	(48.7)	97.5 (12.5)	99.5 (4.5)	97.6 (10.4)
Neuroblastoma	69	40 (58.0)	29 (42.0)	39 (56.5)	30 (43.5)	36 (52.2)	33 (47.8)	98.6 (13.9)	99.7 (5.0)	98.3 (10.7)
Retinoblastoma	38	25 (65.8)	13 (34.2)	23 (60.5)	15 (39.5)	21 (55.3)	17 (44.7)	100.1 (11.2)	100.6 (5.3)	99.3 (9.6)
Wilms' tumour	52	25 (48.1)	27 (51.9)	20 (38.5)	32 (61.5)	30 (57.7)	22 (42.3)	98.7 (13.6)	99.2 (4.2)	98.4 (10.6)
Rhabdomyosarcoma	27	13 (48.2)	14 (51.8)	9 (33.3)	18 (66.7)	16 (59.3)	11 (40.7)	101.3 (10.7)	99.5 (3.6)	101.3 (9.5)

non-CNS=non-central nervous system; POBW=proportion of optimal birth weight; POBL=proportion of optimal birth length; POWFL=proportion of optimal weight for length.

**Table 2:** Cox univariate regression analysis of non-CNS solid tumours in children aged 0–14 years in Western Australia

	**Female sex**		**Not first born**		**POBW z-score**		**POBL z-score**		**POWFL z-score**	
	**HR**	**CI**	**HR**	**CI**	**HR**	**CI**	**HR**	**CI**	**HR**	**CI**
Neuroblastoma	0.75	(0.47,1.22)	0.98	(0.61,1.57)	1.09	(0.86,1.38)	1.04	(0.82,1.32)	1.07	(0.85,1.36)
Retinoblastoma	0.54	(0.28,1.06)	0.86	(0.46,1.64)	1.22	(0.90,1.66)	1.26	(0.92,1.72)	*1.18*	(0.86,1.60)
Wilms' tumour	1.12	(0.65,1.94)	0.80	(0.46,1.39)	1.10	(0.84,1.44)	0.93	(0.71,1.22)	1.08	(0.82,1.42)
Rhabdomyosarcoma	1.12	(0.53,2.38)	0.75	(0.35,1.62)	1.33	(0.93,1.90)	0.99	(0.68,1.44)	1.41	(0.98,2.01)

HR=hazard Ratio; CI=95% confidence interval; POBW=proportion of optimal birth weight; POBL=proportion of optimal birth length; POWFL=proportion of optimal weight for length.
